# Transcription factor FTZ-F1 regulates mosquito cuticular protein CPLCG5 conferring resistance to pyrethroids in *Culex pipiens pallens*

**DOI:** 10.1186/s13071-020-04383-w

**Published:** 2020-10-14

**Authors:** Yang Xu, Xiaoshan Yang, Xiaohong Sun, Xixi Li, Zhihan Liu, Qi Yin, Lei Ma, Dan Zhou, Yan Sun, Bo Shen, Changliang Zhu

**Affiliations:** 1 Department of Pathogen Biology, Nanjing Medical University, Nanjing, China; 2 Department of Blood Transfusion, The First Affiliated Hospital of Nanjing Medical University, Nanjing, China

**Keywords:** *Culex pipiens pallens*, Deltamethrin, Resistance, Cuticular protein, CPLCG5, Transcription factor, FTZ-F1

## Abstract

**Background:**

*Culex pipiens pallens* poses a serious threat to human health because of its widespread distribution, high carrier capacity for several arboviruses, frequent human-biting, and growth in urban environments. Pyrethroid insecticides have been mainly used to control adult *Cx. pipiens pallens* during outbreaks of mosquito-borne diseases. Unfortunately, mosquitoes have developed resistance, rendering the insecticides ineffective. Cuticular resistance is the primary mechanism of pyrethroid resistance. Previously, we revealed that cuticular protein of low complexity CPLCG5 is a major cuticular protein associated with deltamethrin resistance in *Cx. pipiens pallens*, which is enriched in the cuticle of mosquitoes’ legs and participates in pyrethroid resistance by forming a rigid matrix. However, the regulatory mechanisms of its transcription remain unknown.

**Results:**

First, qRT-PCR analysis revealed that the expression of FTZ-F1 (encoding Fushi tarazu-Factor 1) was ~ 1.8-fold higher in the deltamethrin-resistant (DR) than deltamethrin-susceptible (DS) strains at 24 h post-eclosion (PE) and ~ 2.2-fold higher in the DR strain than in the DS strain at 48 h PE. *CPLCG5* and *FTZ-F1* were co-expressed in the legs, indicating that they might play an essential role in the legs. Dual luciferase reporter assays and EMSA (electrophoretic mobility shift experiments) revealed that FTZ-F1 regulates the transcription of *CPLCG5* by binding to the FTZ-F1 response element (− 870/− 864). Lastly, knockdown of *FTZ-F1* not only affected *CPLCG5* expression but also altered the cuticle thickness and structure of the legs, increasing the susceptibility of the mosquitoes to deltamethrin *in vivo.*

**Conclusions:**

The results revealed that FTZ-F1 regulates the expression of CPLCG5 by binding to the *CPLCG5* promoter region, altering cuticle thickness and structure, and increasing the susceptibility of mosquitoes to deltamethrin in vivo. This study revealed part of the mechanism of cuticular resistance, providing a deeper understanding of insecticide resistance.
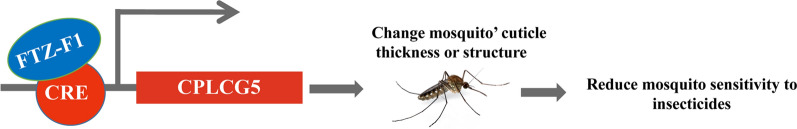

## Background

Mosquitoes of the *Cx. pipiens* complex are widely distributed and spread many human diseases. *Culex pipiens pallens* is the primary vector of the filamentous nematode, *Wuchereria bancrofti*, which is widely prevalent in Asia and sub-Saharan Africa. *Wuchereria bancrofti* can block the lymphatic system and lead to elephantiasis and hydrocele, and is considered as one of the leading causes of long-term disability worldwide [1–4]. *Culex pipiens* mosquitoes are also involved in the transmission of other pathogens, such as West Nile virus (WNV), avian malarias, and avian pox virus [5–7]. Insecticides, especially pyrethroids, remain the mainstay to control these important vectors. Unfortunately, resistance to insecticides is now widespread and is increasing rapidly in intensity in *Culex* mosquitoes across China, which have threatened the effectiveness of insecticides and become the major obstacle for mosquito control [8]. A key challenge is to maintain the efficacy of current interventions under the threat of growing insecticide resistance.

To date, three mechanisms related to insecticide resistance have been proposed. Target site and metabolic resistance have been studied extensively. However, little is known about the other mechanism, cuticular resistance. Cuticular resistance involves reducing the penetration of insecticides into the insect body by increasing the cuticle thickness or changing the cuticle composition [9–13]. Indeed, in *Anopheles gambiae* the thickness of the cuticle correlated positively with permethrin metabolism [13]. In addition, cuticle thickening and low permeability of insecticides were observed in deltamethrin resistant *Heliothis armigera* and *Drosophila melanogaster* [14, 15]*.* The cuticle is the first barrier that protects insects from insecticides [16]. Insect cuticles are divided into the epicuticle and the procuticle. The epicuticle forms the outermost layer of the cuticle, which is mainly responsible for water impermeability. The procuticle forms the bulk of the cuticle and consists of the exocuticle and the endocuticle. Cuticular proteins (CPs) are structural proteins, which is widely distributed in the procuticle of insects [17–20]. Recent studies have shown that CPs contribute to the maintenance and structure of the cuticle. Silencing specific CP genes could cause thinner and/or malformed cuticles [21, 22]. It was reported that the cuticular protein of low complexity CPLCG family genes of *An. gambiae* are mainly expressed in adult mosquitoes, are highly expressed in a variety of insecticide-resistant mosquitoes, and are enriched in the endocuticle of the mosquitoes’ legs and antennae [9]. Our previous study found that CPLCG5 was expressed higher in the femur cuticle in the DR strain compared with that in the DS strain [22]. However, the regulatory mechanisms of *CPLCG5* transcription remain unknown. Therefore, it is critical to identify and characterize the transcription factors involved in the regulation of cuticle genes to better understand the underlying mechanism.

**Fig. 1:**
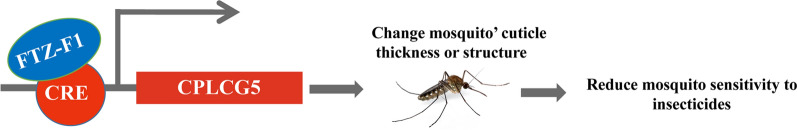
The mode of regulation of transcription factor FTZ-F1 on *CPLCG5* expression

Transcription factors have a significant impact on insect gene expression, morphological diversification, and developmental mechanisms [23]. Fushi tarazu-Factor 1 (FTZ-F1) is a member of the nuclear hormone receptor superfamily and was originally identified in *Drosophila* [24]. Recently, several studies have found that transcription factor FTZ-F1 may be involved in the regulation of insect cuticular proteins. For example, silencing *FTZ-F1* affected the expression of certain cuticle genes in *Apis mellifera* [25]. A cuticle gene, *EDG84A*, is regulated by *FTZ-F1* during the metamorphosis of *D. melanogaster* [26]*.* As for studies of FTZ-F1 regulating insecticide resistance, only a study by Li et al*.* [27] observed that FTZ-F1 mediates the expression of *CYP6BG1*, conferring resistance to chlorantraniliprole in *Plutella xylostella.* However, the relationship between FTZ-F1 and cuticular resistance has not been reported. Our transcriptome study suggested that the gene *FTZ-F1* is highly expressed in the DR strain, and there was a predicted FTZ-F1 binding site in the *CPLCG5* promoter region. Does FTZ-F1 regulate the expression of *CPLCG5* by binding to the promoter region of *CPLCG5*, causing altered cuticle thickness and structure, and thus affecting insecticide resistance in mosquitoes (Fig. 1)? The present study aimed to explore the regulatory role of FTZ-F1 in deltamethrin resistance by regulating the expression of *CPLCG5*.

## Methods

### Mosquito strains

The DS strain of *Cx. pipiens pallens* (LC_50_ = 0.03 mg/l) was collected from Tangkou (Shandong province, China) and maintained in our laboratory without exposure to any insecticides. The DR strain was from the DS strain by repeated selection for 84 generations at the larval stage, and the LC_50_ was up to 7.5 mg/l. Other details of the strains have been described previously [22].

### RNA extraction, cDNA synthesis, and quantitative real-time reverse transcription PCR (qRT-PCR)

Total RNA was extracted from 10 female mosquitoes, or tissues of 25 mosquitoes, per tube (3 replicates). The detailed method of RNA extraction and cDNA synthesis was described previously [28]. The primers used for qRT-PCR are listed in Additional file 1: Table S1. The qRT-PCR experiment was performed using a previously described method [28]. *ACTB* (encoding β*-*actin) was used as an internal control [29, 30]. The 2^−ΔΔCt^ method was used to analyze the data [31].

### Gene silencing

The small interfering RNA (siRNA) targeting *FTZ-F1* (siFTZ-F1), and the negative control (siNC) were designed and synthesized by GenePharma (Shanghai, China; Additional file 1: Table S1). Approximately 364 ng of siFTZ-F1, 350 ng of siNC, and 0.07 μl of diethyl pyrocarbonate (DEPC) water-negative control were injected into the thorax of female mosquitoes in the DR strain, separately. Other details of the injection method have been described previously [28]. Subsequently, the injected mosquitoes were allowed to recover under standard rearing conditions for further investigation. At 3 days post-injection, qRT-PCR was used to determine the gene silencing efficiency. The remaining mosquitoes were then selected for subsequent experiments.

### Western blot analysis

Samples (10 mosquitoes per tube, 3 replicates) were homogenized in radioimmunoprecipitation assay lysis buffer (Beyotime, Jiangsu, China) containing the protease inhibitor phenylmethylsulfonyl fluoride. After centrifugation (3200×*g* for 10 min at 4 °C), the pellet was discarded, and the supernatant was analyzed using sodium dodecyl sulfate polyacrylamide gel electrophoresis and western blotting. Western blotting was performed as described previously [28]. An anti-β-actin monoclonal antibody (1:2000; Cell Signaling Technology, Danvers, MA, USA) was used as the internal control and the anti-CPLCG5 (1:1500) polyclonal antibody was custom made [22].

### Centers for Disease Control and Prevention (CDC) bottle bioassay

The CDC bottle bioassay has been described previously [22]. In each bottle, approximately 20 4-day-old non-blood-fed female mosquitoes from the siFTZ-F1, siNC, and DEPC water groups were introduced into bottles coated with deltamethrin (7.5 mg/ml) and incubated for 2 h. An acetone-coated bottle served as the no insecticide control. Mortality was assessed every 15 min during the exposure period. Three replicates were performed for each group.

### Full-length cloning of *FTZ-F1*

The full-length cDNA of *FTZ-F1* from *Cx. pipiens pallens* was amplified in two sections using 5′- and 3′-rapid amplification of cDNA ends (RACE). The 5′- and 3′-RACE products were obtained using a SMARTer® RACE 5′/3′ Kit (Takara, Shiga, Japan). We assembled the two sections to generate the full-length cDNA. Then, the open reading frame (ORF) was amplified according to the sequence of the putative full-length cDNA. All primer sequences for RACE and ORF amplification are presented in Additional file 2: Table S2).

### Cloning and computer-based analysis of the promoter region of *CPLCG5*

The upstream 1.744 kb promoter region of the *CPLCG*5 gene (GenBank: KF723314) was cloned using PCR and analyzed using the JASPAR program (https://jaspar.genereg.net/) to identify putative response elements. The sequence of the FTZ-F1-binding site is 5′-TTA ATG A-3′ [33]. The transcription start site was predicted using the promoter predictor NNPP v. 2.2 (https://www.fruitfly.org/seq_tools/promoter.html).

### Vector construction and luciferase assay

The upstream 1.744 kb regulatory region of *CPLCG*5 was cloned into a pGL3-basic Firefly luciferase reporter vector (Promega, Madison, WI, USA) to generate CPLCG5-pGL3-basic. The ORF of *FTZ-F1* was cloned into pEGFP-N1 (EGFP, enhanced green fluorescent protein) (Solarbio, Beijing, China) to generate FTZ-F1-EGFP. Single mutation of the putative response elements was performed using a QuickMutation™ Kit (Beyotime) using the CPLCG5-pGL3-basic plasmid as a template. Mutation positions of the FTZ-F1 binding site are shown in Fig. 5a.

*Drosophila* S2 cells were maintained at 28 °C in *Drosophila* medium (Gibco, Grand Island, NY, USA) supplemented with 10% fetal bovine serum (Gibco). Cell transfections were conducted using the Effectene® Transfection Reagent (Qiagen, Hilden, Germany). Endotoxin-free plasmid DNA (0.2 μg of the constructs and 0.02 μg of pRL-TK) was mixed with 5 μl of Effectene®Transfection Reagent according to the manufacturer’s instructions. After 48 h of transfection, the cells were lysed and subjected to a luciferase assay performed under the Dual Luciferase Reporter Assay System (Promega).

### Electrophoretic mobility shift assay (EMSA)

To test the binding of FTZ-F1 to regulatory sequences in the *CPLCG5* promoter, an EMSA experiment was performed using a LightShift Chemiluminescent EMSA Kit (Thermo Fisher Scientific, Waltham, MA, USA). The potential FTZ-F1-binding sequence from *CPLCG5* (− 870 to − 864) was used as a probe and labeled with biotin at the 5′-end. The sequences of the cold probes were the same as those of the labeled probes. Labeled probes and mutant probes were used as competitors for each other. The probes were synthesized by Invitrogen (Shanghai, China). A nuclear extract was obtained by using the NE-PER® Nuclear and Cytoplasmic Extraction Reagents Kit (Thermo Fisher Scientific) according to the manufacturer’s instructions. Membranes were made using the LightShift Chemiluminescent EMSA Kit (Thermo Fisher Scientific) according to the manufacturer’s protocol. The probe and nuclear protein were loaded onto 6.5% polyacrylamide gel and electrophoresed at 100 V for 60 min.

### Immunofluorescence (IF) analysis

Paraffin sections were made from the legs of 4-day-old-DR strain female mosquitoes that were injected with siFTZ-F1 (*n* = 25) or siNC (*n* = 25) at 12 h PE. Tissues were incubated with anti-CPLCG5 antibodies (1:1500 in 2% bovine serum albumin in phosphate-buffered saline-Tween-20). Other detailed steps for IF have been described previously [22]. The fluorescence intensity of pictures were analyzed using Image-Pro Plus (IPP) software.

### Scanning electron microscope (SEM)

To accommodate an effect of body size on cuticle thickness, wing length provides a useful reference for body size [34]. The protocol for SEM was the same as that described by Wood et al. [35]. The cuticle thickness was determined from the SEM images using Image J software (NIH, Bethesda, MD, USA). The mean cuticle thickness per leg were made by tracing the circumferences of both the inner and outer circles of the cuticle and measuring the distance between the two for at least 16 different points.

### Transmission electron microscopy (TEM)

The apical regions of the tarsi leg segment of 8 female mosquitoes from the siFTZ-F1 and siNC groups were dissected. The protocol for TEM was the same as that described by Huang et al*.* [22]. Observation was carried out using a JEM-1210 transmission electron microscope (JEOL, Peabody, MA, USA) at 80 kV.

### Statistical analysis

Mosquito mortality was analyzed using the Chi-square test [36, 37]. Other experimental data were analyzed using the Student’s t-test. All data were presented as the mean ± SD. A value of *P* < 0.05 was considered statistically significant. All experiments were performed using at least 3 independent cohorts.

## Results

### Transcriptome data shows high expression of the nuclear hormone receptor *FTZ-F1* in the DR strain

**Fig. 2:**
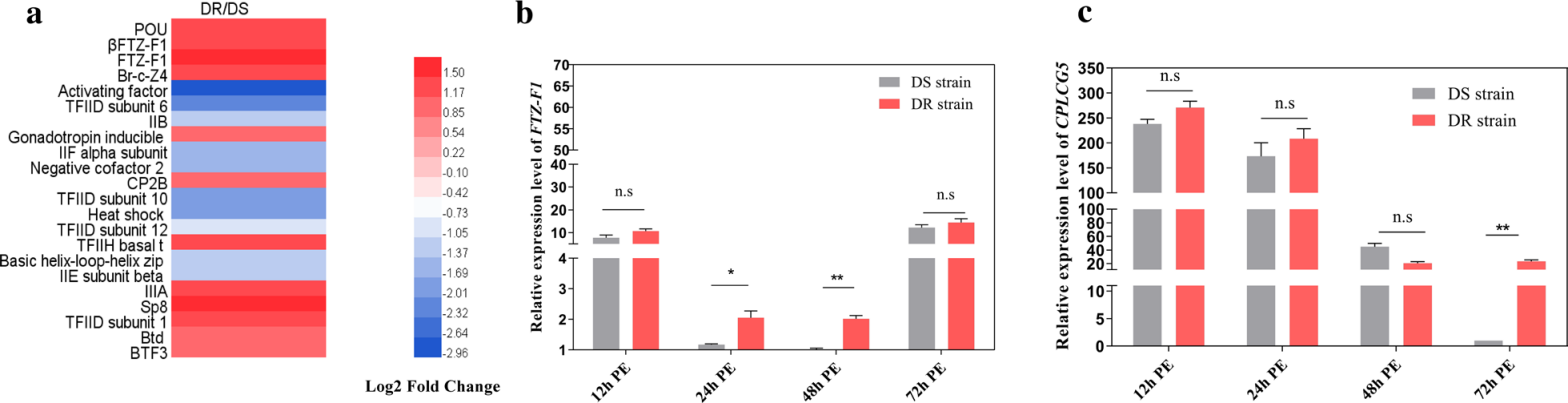
The expression of *CPLCG5* and *FTZ-F1* in DR and DS strains. **a** Heat map of all transcription factors showing their regulation (fold change in expression, resistant *vs* susceptible: DR/DS). Red indicates increased abundance, while green indicates decreased abundance. White indicates no significant change. **b** Expression pattern of *FTZ-F1* in mosquitoes at different developmental stages, as assessed using qRT-PCR. **c** Expression pattern of *CPLCG5* in mosquitoes at different developmental stages, as assessed using qRT-PCR. Relative expression levels were calculated based on the lowest expression value, which was ascribed an arbitrary value of 1. Results are shown as the mean ± SD of 3 biological replicates. *Abbreviation*: PE, post-eclosion. *P ≤ 0.05; **P ≤ 0.01; ns, not significant (*P* > 0.05)

A detailed heat map in Fig. 2a shows the differential expression levels of all transcription factors (TFs) detected in mosquitoes. The *FTZ-F1* gene showed the greatest difference in expression between the DR and DS strains among all TFs (1.8-fold, t-test: *t*_(4)_ = 4.485, *P* = 0.0007). The results suggested that *FTZ-F1* might be related to insecticide resistance.

### Temporal expression patterns of *FTZ-F1* and *CPLCG5 *in the DR and DS strains of *Cx. pipiens pallens*

To examine the role of *FTZ-F1* and *CPLCG5* in the resistance in *Cx. pipiens pallens*, we detected their expression patterns at different developmental stages from female mosquitoes at several time points. Analysis using qRT-PCR revealed that the expression of *FTZ-F1* was different at 24 and 48 h PE between the DR and DS strains, 1.8-fold (t-test: *t*_(4)_ = 3.920, *P* = 0.0172) and 2.2-fold (t-test: *t*_(4)_ = 8.527, *P* = 0.0010), respectively (Fig. 2b), and the expression of *CPLCG5* showed the most significant difference at 72 h PE between the DR and DS strains (Fig. 2c). This finding further suggested that overexpression of *FTZ-F1* might be related to deltamethrin resistance.

### *CPLCG5* and *FTZ-F1* are highly expressed in mosquito legs

**Fig. 3:**
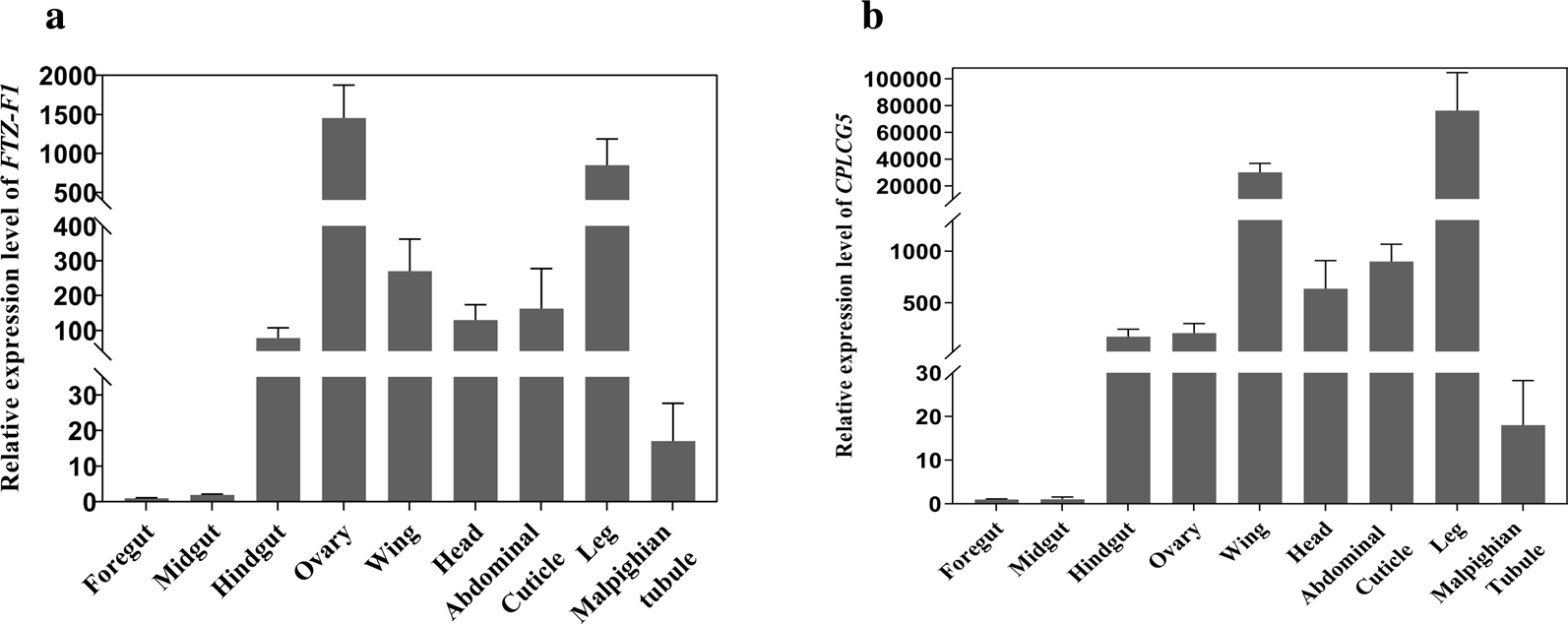
Expression profiles of *FTZ-F1* and *CPLCG5* in different mosquito tissues. **a** Constitutive expression of *FTZ*-*F1* in DR strain. **b** Constitutive expression of *CPLCG5* in the DR strain. mRNA expression levels in the foregut, midgut, hindgut, ovary, wing, head, abdominal cuticle, legs and Malpighian tubules in the DR strain. Relative expression levels were calculated based on the lowest expression value, which was ascribed an arbitrary value of 1. Results are shown as the mean ± SD of 3 biological replicates

We examined the expression of *FTZ-F1* and *CPLCG5* in different tissues from female mosquitoes at 72 h PE using qRT-PCR. The results revealed that *FTZ-F1* was highly expressed in the ovaries and legs (Fig. 3a) and *CPLCG5* was highly expressed in the wings and legs (Fig. 3b), which indicated that *FTZ-F1* and *CPLCG5* might play important roles in mosquito legs.

### Enhanced expression of CPLCG5 by FTZ-F1

**Fig. 4:**
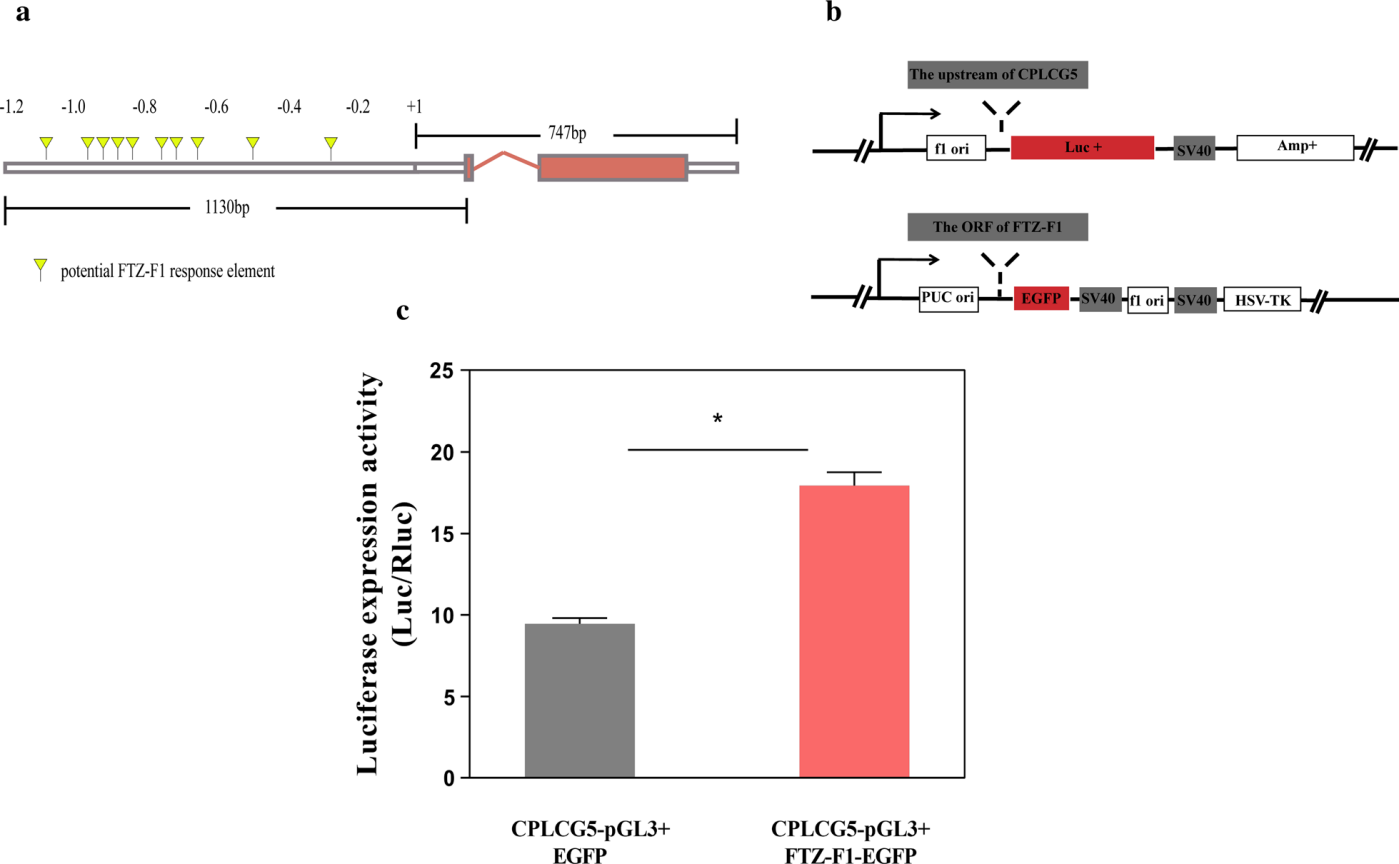
Enhanced expression of CPLCG5 by FTZ-F1. **a** Predicting the binding sites of FTZ-F1 in the *CPLCG5* promoter region. **b** Schematic diagram of the vector construction; the upstream 1.744 kb regulatory region of the *CPLCG*5 gene was cloned into the pGL3-basic Firefly luciferase reporter vector to generate CPLCG5-pGL3-basic (B, top). The ORF of *FTZ-F1* was cloned into a pEGFP-N1 to generate FTZ-F1-EGFP (B, bottom). **c** Activity assay of the luciferase reporter in S2 cells co-transfected with CPLCG5-pGL3-basic vector and EGFP vector or CPLCG5-pGL3-basic and FTZ-F1-EGFP vector. The results are shown as the mean ± SD of 3 biological replicates. **P ≤ 0.01

FTZ-F1 is a TF with a DNA-binding domain. We predicted 10 potential binding sites using the FTZ-F1 recognition sequence (TTA ATG A) in the JASPAR website (Fig. 4a). FTZ-F1-EGFP vector and CPLCG5-pGL3-Luc vector were constructed using the method shown in Fig. 4b. The expression levels of the luciferase gene under the control of the *CPLCG5* promoter increased by 1.8-fold over the controls in cells overexpressing FTZ-F1 (Fig. 4c, t-test: *t*_(4)_ = 16.47, *P* = 0.0063). These results demonstrated that FTZ-F1 upregulated the *CPLCG5* expression by binding to its promoter region.

### Mutation of 10 potential binding sites

**Fig. 5:**
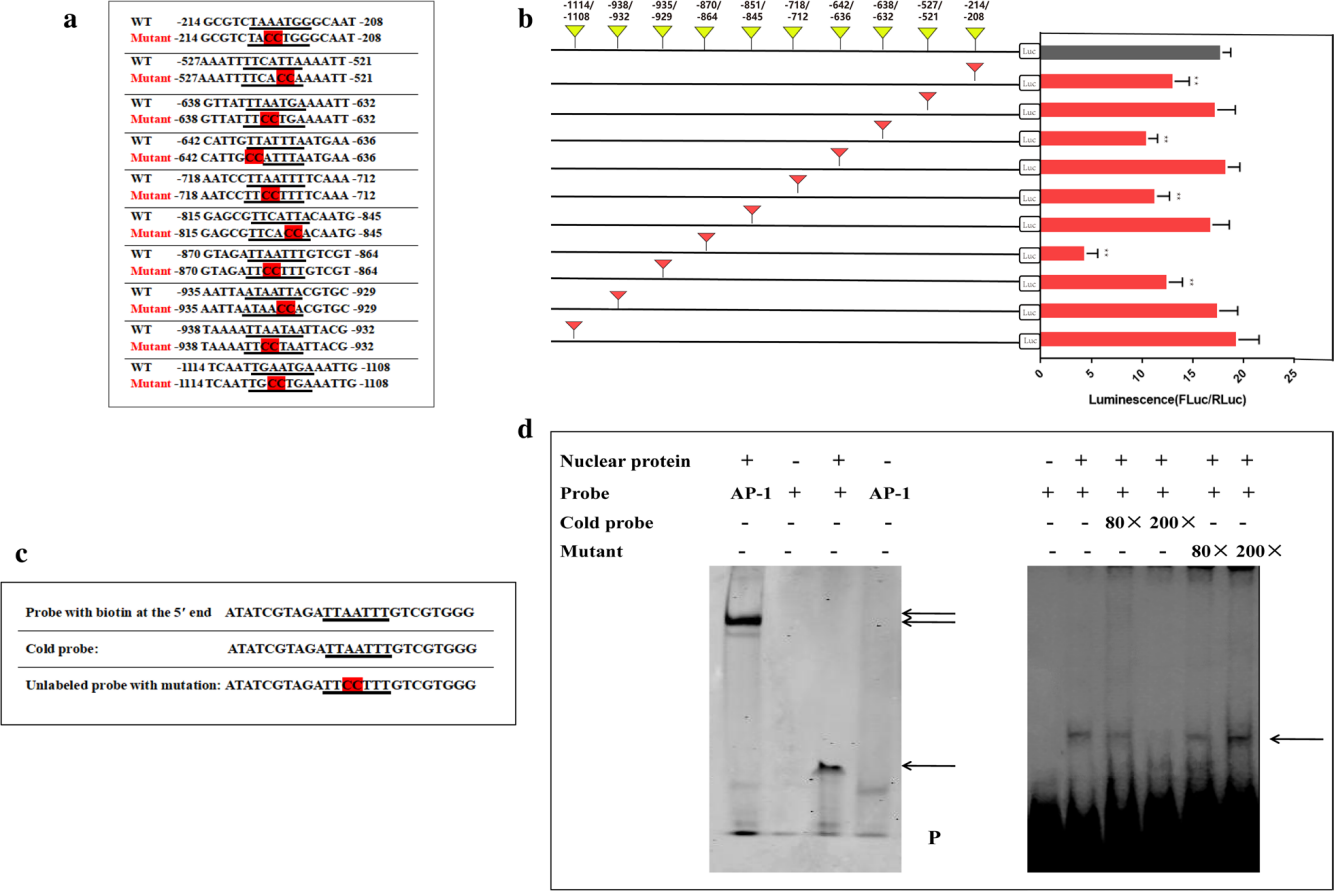
Identification of binding sites for FTZ-F1 in the *CPLCG5* promoter region. **a** Mutation positions of the FTZ-F1 binding site. The red mark indicates the region of mutation. **b** The effect of *CPLCG5* promoter activity of mutating the FTZ-F1 binding site. **c** Probes for EMSA. **d** Analysis of the FTZ-F1 response element on the *CPLCG5* promoter by EMSA. The results are shown as the mean ± SD of 3 biological replicates. *P ≤ 0.05

To identify the key region and the core elements required for *CPLCG5* transcription, 10 potential cis-regulating elements (CREs) of *CPLCG5* that might bind to FTZ-F1 were mutated separately using CPLCG5-PGL3 as a template. The TT or AA of the core nucleotide were mutated to CC, and the mutation region is marked in red in Fig. 5a. The result showed that the luciferase activity of the mutated site -870/-864 decreased most significantly, by 76% (t-test: *t*_(4)_ = 17.00, *P* = 0.0120), compared with that of the wild-type CPLCG5-pGL3-Luc construct (Fig. 5b), indicating that -870/-864 of *CPLCG5* might be the main cis-regulating element for FTZ-F1 to regulate *CPLCG5* expression.

### EMSA experiments

The labeled probes, cold probes, and mutated probes were designed according to the sequence of the main cis-regulatory element -870/-864 (Fig. 5c). Ten mosquito nucleoproteins were used in the EMSA binding reaction and a specific shifted band was detected (Fig. 5d). Furthermore, the specificity of the binding was confirmed by competition experiments, which showed that the signal was competed for by the cold probes. When the competitive probes were added at 200-times excess, we could hardly detect the shifted band (Fig. 5d). When we mutated the critical nucleotides of the probe, as shown in Fig. 5c, the signal of the band did not shift (Fig. 5d). These results suggested that the binding site -870/-864 is the main CRE through which FTZ-F1 regulates *CPLCG5* expression.

### Silencing of FTZ-F1 affects the expression of CPLCG5

**Fig. 6:**
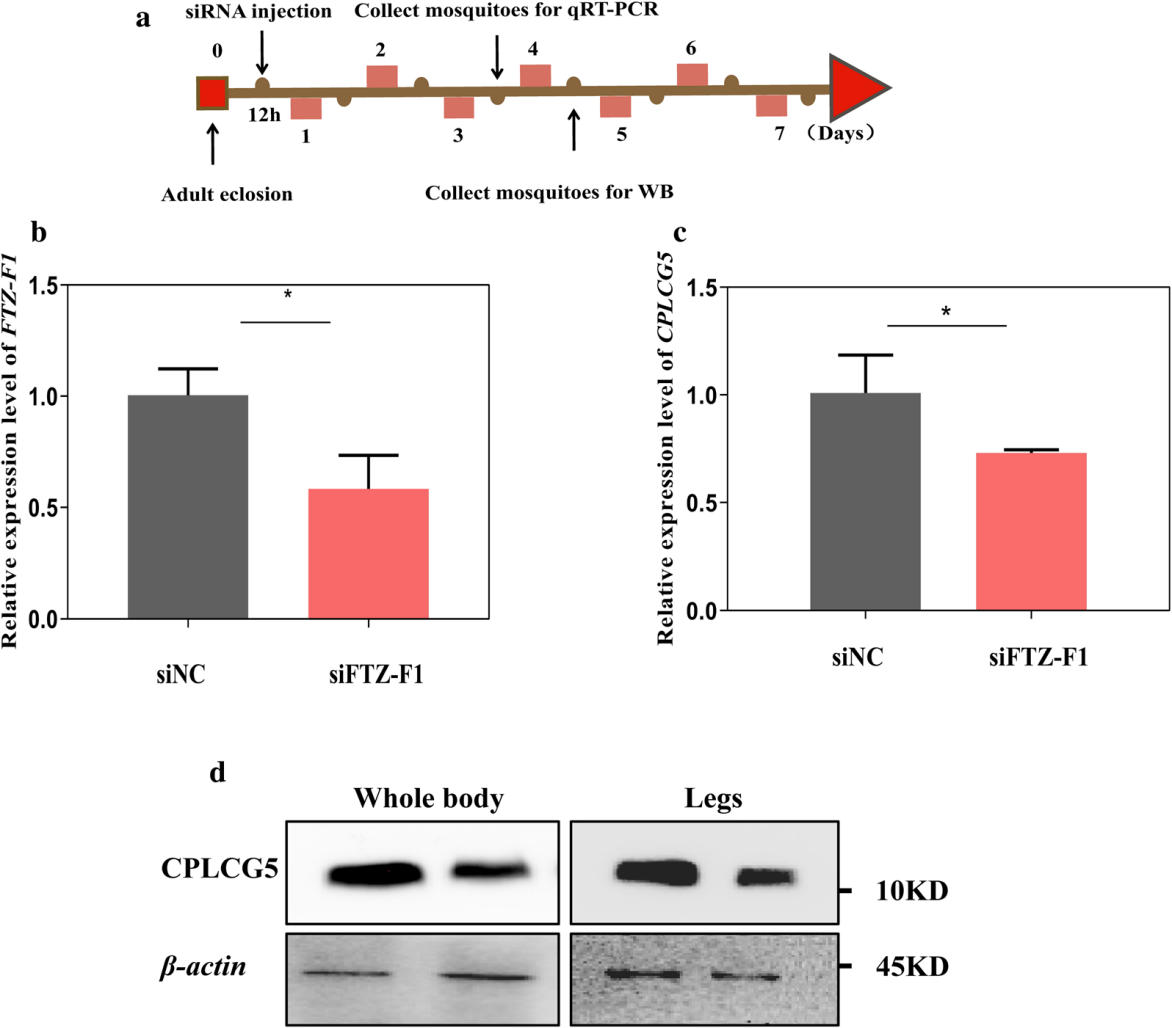
Relative expression levels of FTZ*-*F1 and CPLCG5 after injection of siFTZ-F1. **a** Schematic diagram of the experimental time course for siRNA microinjection, qRT-PCR, and western blotting. **b** Relative expression level of *FTZ-F1* after injection of siFTZ-F1, as assessed using qRT-PCR. **c** Relative expression level of *CPLCG5* after injection of siFTZ-F1, as assessed using qRT-PCR. **d** The levels of CPLCG5 in whole mosquito bodies and legs after silencing of *FTZ-F1*, as assessed using western blotting. *n* = 10 mosquitoes per tube. The results are shown as the mean ± SD of 3 biological replicates. *P ≤ 0.05

To further determine whether the TF FTZ-F1 regulates the expression of *CPLCG5*, the expression of *FTZ-F1* was silenced by injecting female mosquitoes with an siRNA targeting *FTZ-F1* at 12 h PE (Fig. 6a). The relative expression of *FTZ-F1* was significantly reduced by 42% (t-test: *t*_(2)_ = 9.725, *P* = 0.0104) compared with that in the NC group (Fig. 6b). The expression of *CPLCG5* was suppressed by 30% (Fig. 6c; t-test: *t*_(4)_ = 2.745, *P* = 0.0480) and the level of the CPLCG5 protein decreased by 66% (t-test: *t*_(4)_ = 6.205, *P* = 0.0034) in the whole body of mosquitoes and by 42% (t-test: *t*_(4)_ = 4.852, *P* = 0.0083) in the mosquito legs after silencing of *FTZ-F1* (Fig. 6d, Additional file 3: Figure S1). These results revealed that the expression of *CPLCG5* is regulated by FTZ-F1.

### Role of FTZ-F1 in deltamethrin resistance of mosquitoes

**Fig. 7:**
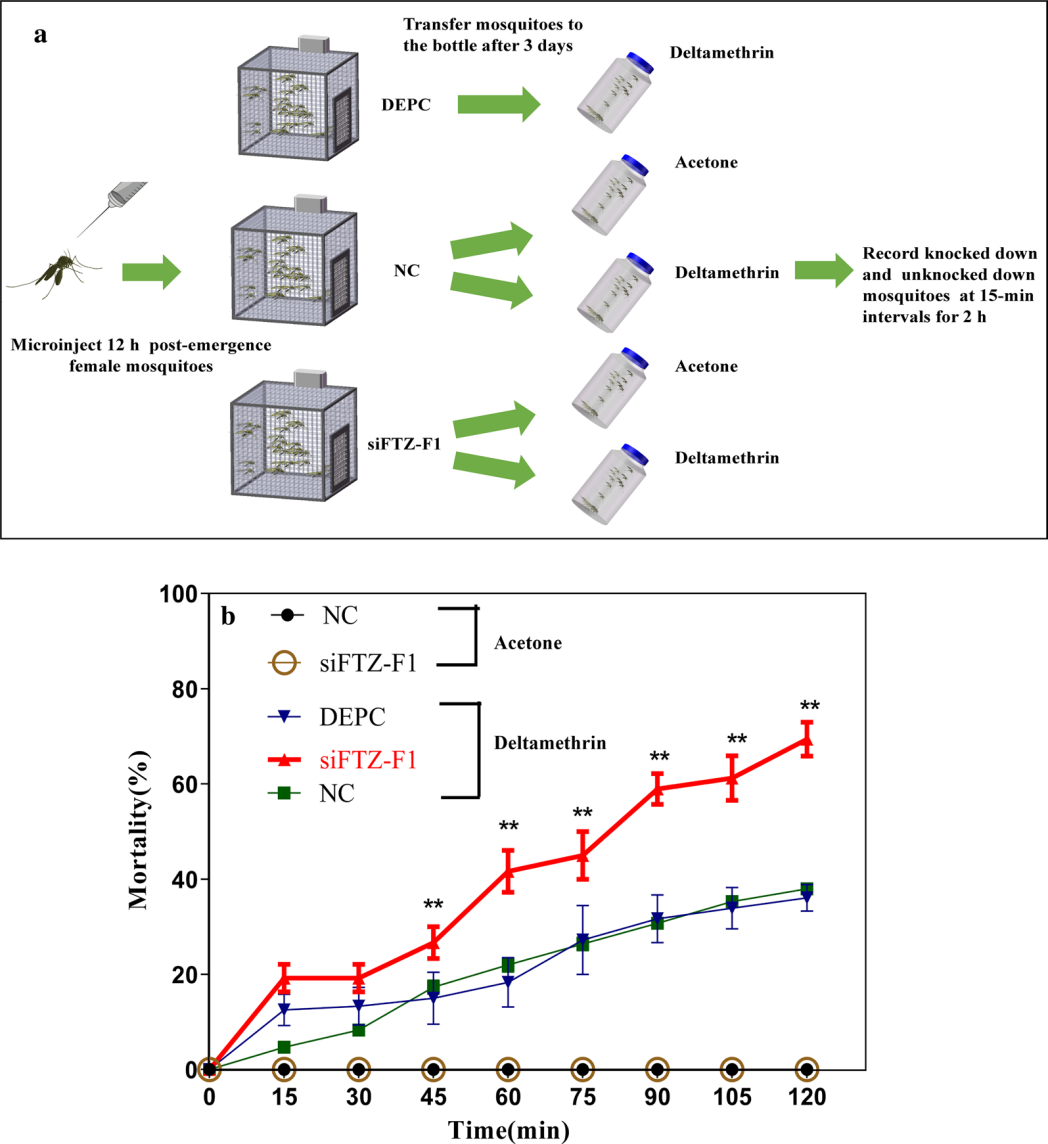
CDC bottle bioassay of *FTZ-F1* knockdown in the DR strain. **a** Schematic of the procedure of CDC bottle bioassay. **b** Insecticide resistance level after silencing of *FTZ-F1*, as analyzed using the CDC bottle assays (7.5 mg/ml). The results are presented as the mean ± SD of 3 independent experiments. Statistical values were calculated relative to the NC group. There was no statistically significant difference between the DEPC and NC injected groups. **P < 0.01

To confirm our hypothesis that FTZ-F1 is involved in deltamethrin resistance, we performed a CDC bottle bioassay (Fig. 7a). The CDC bottle bioassay showed the mortality rate of the siFTZ-F1 group in the DR strain increased at 45, 60, 90, 105 and 120 min compared with that in the control (Fig. 7b), confirming the functional involvement of FTZ-F1 in deltamethrin resistance.

### Silencing of FTZ-F1 changed the expression and distribution of CPLCG5 protein in the leg cuticle

**Fig. 8:**
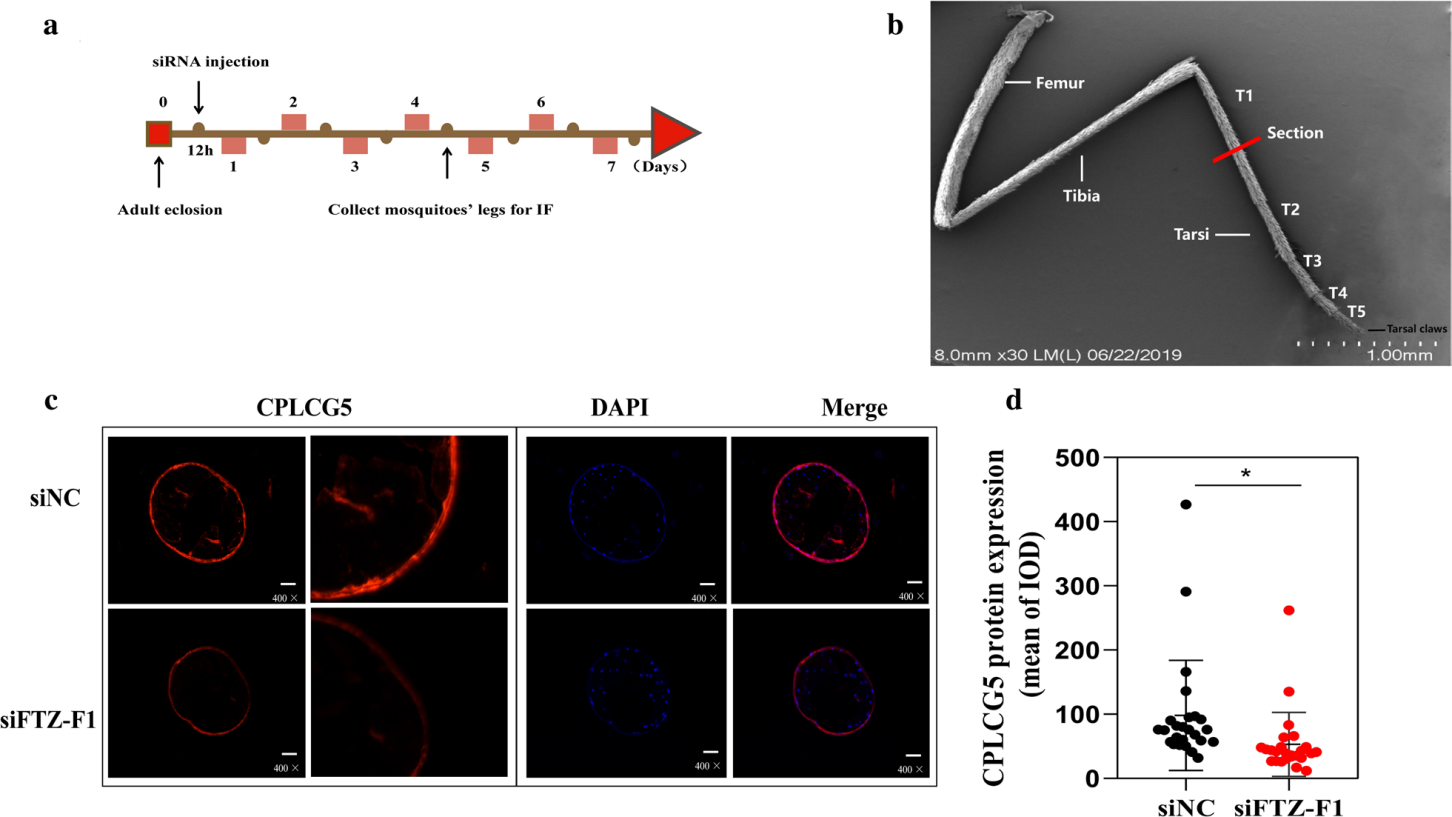
Silencing of *FTZ-F1* reduced the expression of CPLCG5 protein. **a** Schematic diagram of experimental time course for siRNA microinjection and IF. **b** Illustration of the position of sectioning on the *Cx. pipiens pallens* tarsomere 1 (t1-t5 = five tarsal segments [52]). The red line indicates in which leg part the sections were taken. **c** Representative images of CPLCG5 localization in the tarsi of the siNC and siFTZ-F1 groups. Each IF panel comprises three images, with red fluorescence of CPLCG5 on the left, nuclei stained with DAPI in the middle, and merged photos on the right. **d** Quantification of CPLCG5 protein levels in the tarsi of the siNC and siFTZ-F1 groups using Image-Pro Plus (IPP) software. *P < 0.05 indicates a significant difference. Results are shown as the mean ± SD. *n* = the number of measures carried out on each batch of 25 mosquitoes (siNC & siFTZ-F1)

Insecticide contact would be mediated predominately by the tarsi, and hence these most distal leg segments are expected to play the most important role in insecticide uptake. We dissected the tarsi leg segment of female mosquitoes (25 per group), which had been injected with siFTZ-F1 or siNC at 12 h PE (Fig. 8a, b). The results showed that silencing of *FTZ-F1* significantly reduced the level of the CPLCG5 protein, by 46% (t-test: *t*_(48)_ = 2.272, *P* = 0.0276) and resulted the disrupted distribution of CPLCG5 in the mosquito leg cuticle (Fig. 8c, d).

### SEM analysis of cuticle thickness

**Fig. 9:**
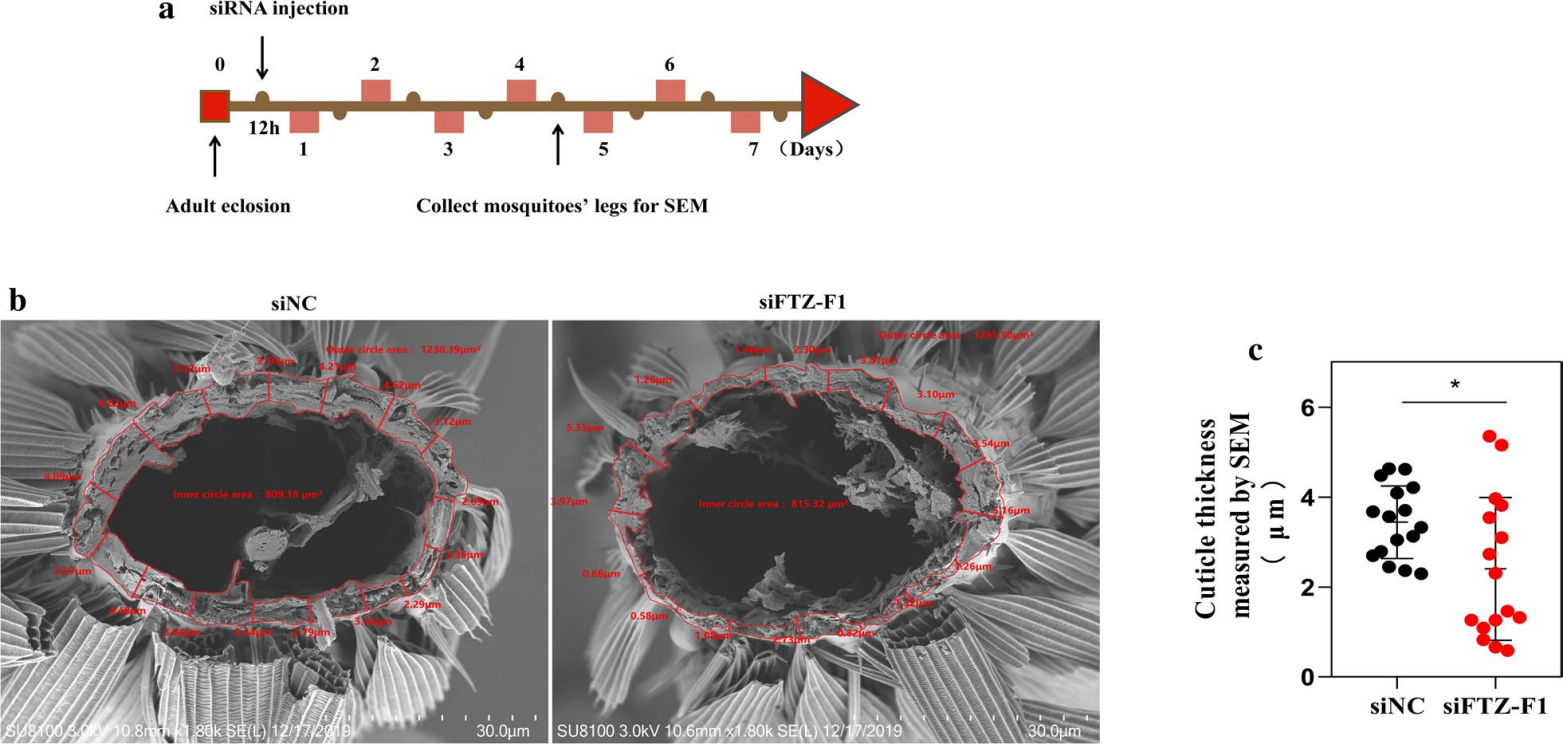
SEM analysis of cuticle thickness. **a** Schematic diagram of the experimental time course for siRNA microinjection and SEM. **b** SEM showing a front view of a sectioned leg of the siNC group and the siFTZ-F1 group. **c** Column bar graph (vertical) of the entire cuticle thickness. 16 points of measurement were used per individual, allowing for calculation of mean cuticle thickness. Results are shown as the mean ± SD. *n* = the number of measures carried out on each batch of 5 mosquitoes (siNC & siFTZ-F1). *P ≤ 0.05

The same number (*n* = 5) of sections were taken in both groups, and measurements in each section were performed randomly (Fig. 9a). The results showed that the cuticle architecture was loose and unevenly distributed in the siFTZ-F1 group compared with that in the siNC group (Fig. 9b). The average cuticle thickness was significantly thinner in the siFTZ-F1 group (2.404 ± 1.53 μm) than in the siNC group (3.443 ± 0.78 μm) (Fig. 9c; t-test: *t*_(30)_ = 2.332, *P* = 0.0266).

### Ultrastructure of tarsi segment cuticles from siFTZ-F1 and siNC mosquitoes by TEM

**Fig. 10:**
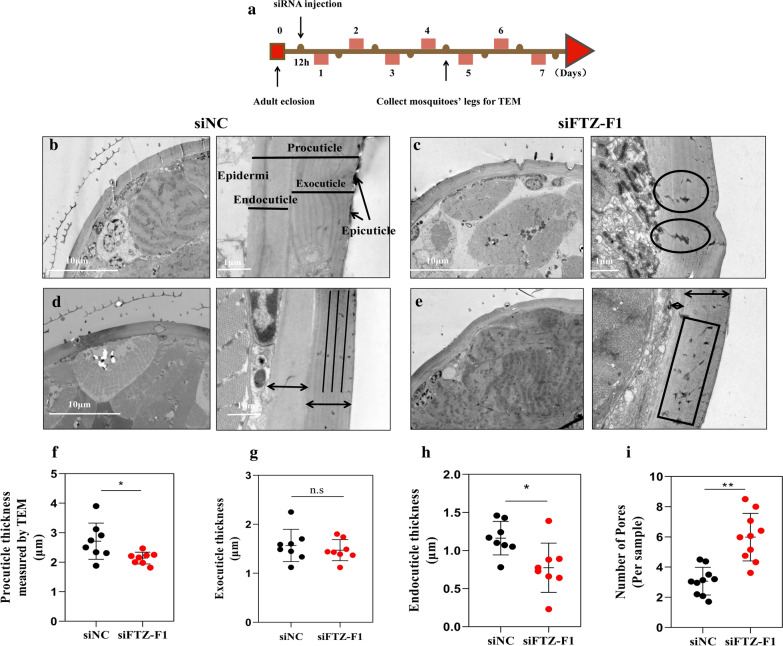
Transmission electron microscopy (TEM) analysis of the ultrastructure of mosquito tarsi cuticles. **a** Schematic diagram of experimental time course for siRNA microinjection and TEM. **b** The ultrastructure of tarsi segment cuticles in siNC group. **c** Enlarged pore canals in siFTZ-F1 group. **d** Distinct chitinous parallel laminae in siNC group. **e** Indistinct chitinous parallel laminae, an increased number of pore canals and thickness measurement of exocuticle and endocuticle in siFTZ-F1 group. The apical regions of the tarsi leg segment of 8 female mosquitoes from the siFTZ-F1 and siNC groups were dissected. Scatter plot of the procuticle thickness (**f**), exocuticle thickness (**g**), endocuticle thickness (**h**), the number of pores (**I**). Results are shown as the mean ± SD. *n* = the number of measures carried out on each batch of 8 mosquitoes (siNC & siFTZ-F1). *P ≤ 0.05; **P ≤ 0.01; ns, not significant (*P* > 0.05)

TEM analysis of tarsi segment of leg cuticles from siFTZ-F1 and siNC mosquitoes (Fig. 10a) suggested that knockdown of *FTZ-F1* resulted in enlarged pore canals (Fig. 10b), indistinct chitinous parallel laminae (Fig. 10c), and an increased number of pore canals (Fig. 10d), in the cuticle structure. The thickness of the overall cuticle was significantly thinner in the siFTZ-F1 group (2.14 ± 0.18 μm) compared with that in the siNC group (2.7 ± 0.57 μm) (Fig. 10e; t-test: *t*_(14)_ = 2.500, *P* = 0.0255), mainly because the endocuticle thickness decreased by 33% in the siFTZ-F1 group compared with that in the siNC group (Fig. 10f, g; t-test: *t*_(14)_ = 2.808, *P* = 0.0140). At the same time, we carried out a TEM analysis of the ultrastructure of mosquito tarsi cuticle for the DR strain and DS strain as a reference (Additional file 4: Figure S2).

## Discussion

The present study showed that FTZ-F1 has a key role in conferring cuticular resistance in *Cx. pipiens pallens* by regulating the expression of *CPLCG5*. Importantly, this study highlights the critical role of FTZ-F1 in mosquito legs, which are main body part that contacts insecticides during mosquito control. The findings provide novel insights into the putative mechanisms of cuticular resistance.

To date, the role of TFs in insecticide resistance has been reported in some studies. For example, in *D. melanogaster*, Nrf2/Maf regulate the expression of the five alleles of *Cyp6a2*, which is involved in DDT resistance [38]. In *Leptinotarsa decemlineata*, the TF CncC is involved in deltamethrin resistance by regulating the expression of *CYP4G7*, *CYP4G14*, *GST-1*, and four ABC transporters [39]. In addition, the TF Maf-S regulates the expression of multiple resistant p450 genes in *An. gambiae*, increasing the susceptibility of mosquitoes to pyrethroid and DDT insecticides [40]. Our previous transcriptome study found that the TF *FTZ-F1* gene was highly expressed in the DR strain, suggesting that FTZ-F1 might be associated with insecticide resistance [32]. FTZ-F1 is a member of the nuclear receptor superfamily [41], and has been cloned in a variety of insects, such as *D. melanogaster* [42], *Bombyx mori* [43], *Aedes aegypti* [44] and *L. decemlineata* [45]. FTZ-F1 has been reported to be related to growth and development. For example, Liu et al*.* [45] found that knockdown of FTZ-F1 in final-instar larvae caused significant impairment of pupation in *L. decemlineata* [45]. In *A. mellifera*, cuticle pigmentation and wing formation during developmental processes were severely impaired by downregulation of FTZ-F1 [25]. Recently, Li et al*.* [27] reported that FTZ-F1 mediates the expression of *CYP6BG1*, conferring resistance to chlorantraniliprole in *P. xylostella.* However, there are no reports on the relationship between FTZ-F1 and cuticular resistance in mosquitoes.

Vannini et al*.* [9] found that the three homologous genes, *CPLCG5*, *CPLCG3* and *CPLCG4*, were expressed at high levels in mosquito legs and wings [9]. Previous research in our laboratory also found that CPLCG5 was abundant in mosquito legs and wings [22]. Moreover, studies have reported that an ecdysone-responsive transcription factor determines the temporal expression of cuticular protein genes in wing discs of *B. Mori* [46]. FTZ-F1 is critical for leg development and silencing *FTZ-F1* in the pupal stage can cause physiological defects in *Drosophila* legs [47, 48]. In our study, we found that *FTZ-F1* and *CPLCG5* are both highly expressed in the legs, which indicated that *FTZ-F1* and *CPLCG5* might exert their functions in mosquito legs. Silencing of *FTZ-F1* resulted in a decrease in *CPLCG5* expression, followed by an increased sensitivity to deltamethrin in mosquitoes, suggesting that FTZ-F1 is associated with mosquito resistance by regulating the expression of *CPLCG5*. Silencing *FTZ-F1* also resulted in disrupting the distribution of CPLCG5 in the leg cuticle, indicating that FTZ-F1 participates in mosquito resistance by regulating the expression and distribution of cuticular protein CPLCG5.

Balabanidou et al*.* [49] reported that insecticide contact would be mediated predominately by the tarsi and these most distal leg segments are expected to play the most important role in insecticide uptake. In our study, SEM observations revealed that the cuticle thickness of the mosquito leg tarsi in the control group was relatively uniform, while the cuticle architecture was loose and unevenly distributed in the siFTZ-F1 group. Additionally, TEM observation revealed that silencing *FTZ-F1* resulted in larger pore canals and increased number of pore canals, which might be beneficial for the penetration of insecticides. Furthermore, our study showed that the endocuticle thickness decreased significantly in the siFTZ-F1 group compared with that in the siNC group, leading to a thinner cuticle. Our study revealed that silencing of *FTZ-F1* reduced the expression of CPLCG5 and made the endocuticle thinner, suggesting that FTZ-F1 mainly affects the expression and distribution of CPLCG5 in endocuticle.

Research on transcription factor binding sites is important to study protein-DNA interactions. The TF FTZ-F1 was reported to bind to a CRE upstream of the cuticular gene *WCP5* to regulate its expression in *B. mori* [50]. FTZ-F1 could also regulate the expression of *EDG84A* by binding to two cis elements of this gene in *D. melanogaster* [26]. A recent study found that FTZ-F1 can regulate the expression of *CYB6PG1* by binding to its promoter region (-562 to -340) in *P. xylostella* [27]. The results of the present study revealed that FTZ-F1 specifically binds to *CPLCG5* through the CRE at -870/-864 to directly regulate the expression of cuticular protein CPLCG5. The resistance level of an organism may be controlled by insecticide resistance genes, whose effectiveness can be modulated by inserting, deleting, or mutating cis-elements. Thus, it is necessary to identify cis-regulatory elements that control the expression of insecticide resistance genes, because these sequences could serve as new targets to screen effective insecticides [39, 51]

## Conclusions

In conclusion, FTZ-F1 regulates the expression of *CPLCG5* by binding to its promoter region, thus altering the cuticle thickness and structure, and increasing mosquitoes’ sensitivity to deltamethrin. This study provides new insights into insects’ cuticular resistance mechanisms and provides a theoretical basis for the development of a mosquito control strategy.

## Supplementary information

**Additional file 1: Table S1.** Primers used for qRT-PCR analysis and siRNA synthesis of *FTZ-F1.*

**Additional file 2: Table S2.** PCR primers used to amplify the full-length *FTZ-F1* and the promoter region of *CPLCG5.*

**Additional file 3: Figure S1. a** The expression analysis of CPLCG5 protein in mosquito’s whole body after silencing of *FTZ-F1*, as assessed using IPP software. **b** The expression analysis of CPLCG5 protein in mosquito’s leg after silencing of *FTZ-F1*, as assessed using IPP software. The results are shown as the mean ± SD of 3 biological replicates. (*P* < 0.01**).

**Additional file 4: Figure S2. a** TEM analysis of the ultrastructure of mosquito tarsi cuticles in the DR strain and DS strain. The black double arrow indicates the cuticle thickness. **b** Scatter plot of the cuticle thickness of the DR strain and DS strain. ***P ≤ 0.001. *Abbreviation*: *n* = the number of measures taken for each batch of 8 mosquitoes (siNC & siFTZ-F1).

## Data Availability

Data supporting the conclusions of this article are included within the article and its additional files. All data are fully available without restriction upon request.

## References

[1] Dreyer G, Norões J, Figueredo-Silva J, Piessens WF (2000). Pathogenesis of lymphatic disease in bancroftian filariasis: a clinical perspective. Parasitol Today.

[2] Krishna Kumari A, Harichandrakumar KT, Das LK, Krishnamoorthy K (2005). Physical and psychosocial burden due to lymphatic filariasis as perceived by patients and medical experts. Trop Med Int Health.

[3] Lima CA, Almeida WR, Hurd H, Albuquerque CMR (2003). Reproductive aspects of the mosquito *Culex quinquefasciatus* (Diptera: Culicidae) infected with *Wuchereria bancrofti* (Spirurida: Onchocercidae). Mem Inst Oswaldo Cruz.

[4] Michael E, Bundy DA, Grenfell BT (1996). Re-assessing the global prevalence and distribution of lymphatic filariasis. Parasitology.

[5] Goddard LB, Roth AE, Reisen WK, Scott TW (2002). Vector competence of California mosquitoes for West Nile virus. Emerg Infect Dis.

[6] Valkiūnas G, Iezhova TA (2018). Keys to the avian malaria parasites. Malar J.

[7] Esteves FCB, Marín SY, Resende M, Silva ASG, Coelho HLG, Barbosa MB (2017). Avian pox in native captive Psittacines, Brazil, 2015. Emerging Infect Dis.

[8] Scott JG, Yoshimizu MH, Kasai S (2015). Pyrethroid resistance in *Culex pipiens* mosquitoes. Pestic Biochem Physiol.

[9] Vannini L, Reed TW, Willis JH (2014). Temporal and spatial expression of cuticular proteins of *Anopheles gambiae* implicated in insecticide resistance or differentiation of M/S incipient species. Parasit Vectors.

[10] Fang F, Wang W, Zhang D, Lv Y, Zhou D, Ma L (2015). The cuticle proteins: a putative role for deltamethrin resistance in *Culex pipiens pallens*. Parasitol Res.

[11] Lilly DG, Latham SL, Webb CE, Doggett SL (2016). Cuticle thickening in a pyrethroid-resistant strain of the common bed bug, *Cimex lectularius* L. (Hemiptera: Cimicidae). PLoS One..

[12] Moussian B (2010). Recent advances in understanding mechanisms of insect cuticle differentiation. Insect Biochem Mol Biol.

[13] Balabanidou V, Kampouraki A, MacLean M, Blomquist GJ, Tittiger C, Juarez MP (2016). Cytochrome P450 associated with insecticide resistance catalyzes cuticular hydrocarbon production in *Anopheles gambiae*. Proc Natl Acad Sci USA.

[14] Ahmad M, Denholm I, Bromilow RH (2006). Delayed cuticular penetration and enhanced metabolism of deltamethrin in pyrethroid-resistant strains of *Helicoverpa armigera* from China and Pakistan. Pest Manag Sci.

[15] Gellatly KJ, Yoon KS, Doherty JJ, Sun W, Pittendrigh BR, Clark JM (2015). RNAi validation of resistance genes and their interactions in the highly DDT-resistant 91-R strain of *Drosophila melanogaster*. Pestic Biochem Physiol.

[16] Noppun V, Saito T, Miyata T (1989). Cuticular penetration of S-fenvalerate in fenvalerate-resistant and susceptible strains of the diamondback moth, *Plutella xylostella* (L.). Pestic Biochem Physiol..

[17] Noh MY, Kramer KJ, Muthukrishnan S, Kanost MR, Beeman RW, Arakane Y (2014). Two major cuticular proteins are required for assembly of horizontal laminae and vertical pore canals in rigid cuticle of *Tribolium castaneum*. Insect Biochem Mol Biol.

[18] Vannini L, Willis JH (2017). Localization of RR-1 and RR-2 cuticular proteins within the cuticle of *Anopheles gambiae*. Arthropod Struct Dev.

[19] Togawa T, Augustine Dunn W, Emmons AC, Willis JH (2007). CPF and CPFL, two related gene families encoding cuticular proteins of *Anopheles gambiae* and other insects. Insect Biochem Mol Biol.

[20] Liao C, Upadhyay A, Liang J, Han Q, Li J (2018). 3,4-Dihydroxyphenylacetaldehyde synthase and cuticle formation in insects. Dev Comp Immunol.

[21] Pan PL, Ye YX, Lou YH, Lu JB, Cheng C, Shen Y (2018). A comprehensive omics analysis and functional survey of cuticular proteins in the *brown planthopper*. Proc Natl Acad Sci USA.

[22] Huang Y, Guo Q, Sun X, Zhang C, Xu N, Xu Y (2018). *Culex pipiens pallens* cuticular protein CPLCG5 participates in pyrethroid resistance by forming a rigid matrix. Parasit Vectors.

[23] Arnosti DN (2003). Analysis and function of transcriptional regulatory elements: insights from *Drosophila*. Annu Rev Entomol.

[24] Yu Y, Li W, Su K, Yussa M, Han W, Perrimon N (1997). The nuclear hormone receptor Ftz-F1 is a cofactor for the *Drosophila* homeodomain protein Ftz. Nature.

[25] Mello TRP, Aleixo AC, Pinheiro DG, Nunes FMF, Cristino AS, Bitondi MMG (2019). Hormonal control and target genes of ftz-f1 expression in the honeybee *Apis mellifera*: a positive loop linking juvenile hormone, ftz-f1, and vitellogenin. Insect Mol Biol.

[26] Murata T, Kageyama Y, Hirose S, Ueda H (1996). Regulation of the *EDG84A* gene by FTZ-F1 during metamorphosis in *Drosophila melanogaster*. Mol Cell Biol.

[27] Li X, Shan C, Li F, Liang P, Smagghe G, Gao X (2019). Transcription factor FTZ-F1 and cis-acting elements mediate expression of *CYP6BG1* conferring resistance to chlorantraniliprole in *Plutella xylostella*. Pest Manag Sci.

[28] Zhou D, Duan B, Xu Y, Ma L, Shen B, Sun Y (2018). NYD-OP7/PLC regulatory signaling pathway regulates deltamethrin resistance in *Culex pipiens pallens* (Diptera: Culicidae). Parasit Vectors.

[29] Hansen IA, Attardo GM, Park JH, Peng Q, Raikhel AS (2004). Target of rapamycin-mediated amino acid signaling in mosquito anautogeny. Proc Natl Acad Sci USA.

[30] Haac ME, Anderson MAE, Eggleston H, Myles KM, Adelman ZN (2015). The hub protein loquacious connects the microRNA and short interfering RNA pathways in mosquitoes. Nucleic Acids Res.

[31] Livak KJ, Schmittgen TD (2001). Analysis of relative gene expression data using real-time quantitative PCR and the 2−ΔΔCt method. Methods.

[32] Lv Y, Wang W, Hong S, Lei Z, Fang F, Guo Q (2016). Comparative transcriptome analyses of deltamethrin-susceptible and -resistant *Culex pipiens pallens* by RNA-seq. Mol Genet Genomics.

[33] Noyes MB, Meng X, Wakabayashi A, Sinha S, Brodsky MH, Wolfe SA (2008). A systematic characterization of factors that regulate *Drosophila* segmentation *via* a bacterial one-hybrid system. Nucleic Acids Res.

[34] Lyimo EO, Takken W (1993). Effects of adult body size on fecundity and the pre-gravid rate of *Anopheles gambiae* females in Tanzania. Med Vet Entomol.

[35] Wood O, Hanrahan S, Coetzee M, Koekemoer L, Brooke B (2010). Cuticle thickening associated with pyrethroid resistance in the major malaria vector *Anopheles funestus*. Parasit Vector.

[36] Zou FF, Guo Q, Sun Y, Zhou D, Hu MX, Hu HX (2016). Identification of protease m1 zinc metalloprotease conferring resistance to deltamethrin by characterization of an AFLP marker in *Culex pipiens pallens*. Parasit Vectors.

[37] Lv Y, Lei Z, Hong S, Wang W, Zhang D, Zhou D (2015). Venom allergen 5 is associated with deltamethrin resistance in *Culex pipiens pallens* (Diptera: Culicidae). J Med Entomol.

[38] Wan H, Liu Y, Li M, Zhu S, Li X, Pittendrigh BR (2014). Nrf2/Maf-binding-site-containing functional Cyp6a2 allele is associated with DDT resistance in *Drosophila melanogaster*. Pest Manag Sci.

[39] Kalsi M, Palli SR (2015). Transcription factors, CncC and Maf, regulate expression of CYP6BQ genes responsible for deltamethrin resistance in *Tribolium castaneum*. Insect Biochem Mol Biol.

[40] Ingham VA, Pignatelli P, Moore JD, Wagstaff S, Ranson H (2017). The transcription factor Maf-S regulates metabolic resistance to insecticides in the malaria vector *Anopheles gambiae*. BMC Genomics.

[41] Lavorgna G, Ueda H, Clos J, Wu C (1991). FTZ-F1, a steroid hormone receptor-like protein implicated in the activation of fushi tarazu. Science.

[42] Yamada M, Murata T, Hirose S, Lavorgna G, Suzuki E, Ueda H (2000). Temporally restricted expression of transcription factor betaFTZ-F1: significance for embryogenesis, molting and metamorphosis in *Drosophila melanogaster*. Development.

[43] Eystathioy T, Swevers L, Iatrou K (2001). The orphan nuclear receptor BmHR3A of *Bombyx mori*: hormonal control, ovarian expression and functional properties. Mech Dev.

[44] Li C, Kapitskaya MZ, Zhu J, Miura K, Segraves W, Raikhel AS (2000). Conserved molecular mechanism for the stage specificity of the mosquito vitellogenic response to ecdysone. Dev Biol.

[45] Liu XP, Fu KY, Lu FG, Meng QW, Guo WC, Li GQ (2014). Involvement of FTZ-F1 in the regulation of pupation in *Leptinotarsa decemlineata* (Say). Insect Biochem Mol Biol.

[46] Ali MS, Iwanaga M, Kawasaki H (2013). Ecdysone-responsive transcriptional regulation determines the temporal expression of cuticular protein genes in wing discs of *Bombyx mori*. Gene.

[47] Broadus J, McCabe JR, Endrizzi B, Thummel CS, Woodard CT (1999). The *Drosophila* beta FTZ-F1 orphan nuclear receptor provides competence for stage-specific responses to the steroid hormone ecdysone. Mol Cell.

[48] Sultan ARS, Oish Y, Ueda H (2014). Function of the nuclear receptor FTZ-F1 during the pupal stage in *Drosophila melanogaster*. Dev Growth Differ.

[49] Balabanidou V, Kefi M, Aivaliotis M, Koidou V, Girotti JR, Mijailovsky SJ (2019). Mosquitoes cloak their legs to resist insecticides. Proc R Soc Lond B Biol Sci.

[50] Wang HB, Nita M, Iwanaga M, Kawasaki H (2009). βFTZ-F1 and broad-complex positively regulate the transcription of the wing cuticle protein gene, BMWCP5, in wing discs of *Bombyx mori*. Insect Biochem Mol Biol.

[51] Kalsi M, Palli SR (2017). Transcription factor cap n collar C regulates multiple cytochrome P450 genes conferring adaptation to potato plant allelochemicals and resistance to imidacloprid in *Leptinotarsa decemlineata* (Say). Insect Biochem Mol Biol.

[52] Evans AM (1938). Mosquitoes of the Ethiopian Region. II.—Anophelini adults and early stages.

