# Supplementary material for: Transcription factor FTZ-F1 regulates mosquito cuticular protein CPLCG5 conferring resistance to pyrethroids in Culex pipiens pallens
Source: Parasit Vectors. 2020 Oct 14;13:514. doi: 10.1186/s13071-020-04383-w (PMC7559895; doi:10.1186/s13071-020-04383-w)
Supplement: Supplementary file 1 — Additional file 1: Table S1. Primers used for qRT-PCR analysis and siRNA synthesis of FTZ-F1. [file 13071_2020_4383_MOESM1_ESM.docx]

**Additional file 1: Table S1**. **Primers used for qRT-PCR analysis and siRNA synthesis of *FTZ-F1***

| Application of primers | Primer name | Primer sequence (5’ to 3’) |
| --- | --- | --- |
| qRT-PCR | FTZ-F1-F | GCGGCAGGTCTCCAGGGTCT |
|  | FTZ-F1-R | AATCGGACTCGGCGAACTGT |
|  | CPLCG5-F | CACCATGCCGGAGTTGTTC |
|  | CPLCG5-R | GGTTCAGTTGCTTCTGGGAC |
|  | β-actin-F | AGCGTGAACTGACGGCTCTTG |
|  | β-actin-R | ACTCGTCGTACTCCTGCTTGG |
| siRNA synthesis | siFTZ-F1-F | GCGCGGAAGCUUCAAAUCATT |
|  | siFTZ-F1-R | UGAUUUGAAGCUUCCGCGCTT |
|  | siNC-F | UUCUCCGAACGUGUCACGUTT |
|  | siNC-R | ACGUGACACGUUCGGAGAATT |

F=Forward; R=Reverse
