# Supplementary material for: Transcription factor FTZ-F1 regulates mosquito cuticular protein CPLCG5 conferring resistance to pyrethroids in Culex pipiens pallens
Source: Parasit Vectors. 2020 Oct 14;13:514. doi: 10.1186/s13071-020-04383-w (PMC7559895; doi:10.1186/s13071-020-04383-w)
Supplement: Supplementary file 2 — Additional file 2: Table S2. PCR primers used to amplify the full-length FTZ-F1 and the promoter region of CPLCG5. [file 13071_2020_4383_MOESM2_ESM.docx]

**Additional file 2: Table S2. PCR primers used to amplify the full-length *FTZ-F1* and the promoter region of *CPLCG5*.**

| Application of primers | Primer name | Primer sequence (5’ to 3’) |
| --- | --- | --- |
| Promter region | CPLCG5-F | GATCGTTTGACATTTCACCA |
|  | CPLCG5-R | TTTGAGTTGGTTGTTTGAGA |
| RACE-FTZ-F1 | 3′ RACE-GSP | GATTACGCCAAGCTTGCTATGGATGAGCGCCAACTC |
|  | 5′RACE-GSP | GATTACGCCAAGCTTGGACTTAGCGAGTGTGGTGAGGC |
| ORF-FTZ-F1 | ORF-F | ACCCAGCAAGAATCCGTCCTA |
|  | ORF-R | ACGAAAATGCCGCCTCCTT |

F=Forward; R=Reverse; GSP : Gene-Specific Primer;
