# Supplementary figures and images for: Transcription factor FTZ-F1 regulates mosquito cuticular protein CPLCG5 conferring resistance to pyrethroids in Culex pipiens pallens
Source: Parasit Vectors. 2020 Oct 14;13:514. doi: 10.1186/s13071-020-04383-w (PMC7559895; doi:10.1186/s13071-020-04383-w)

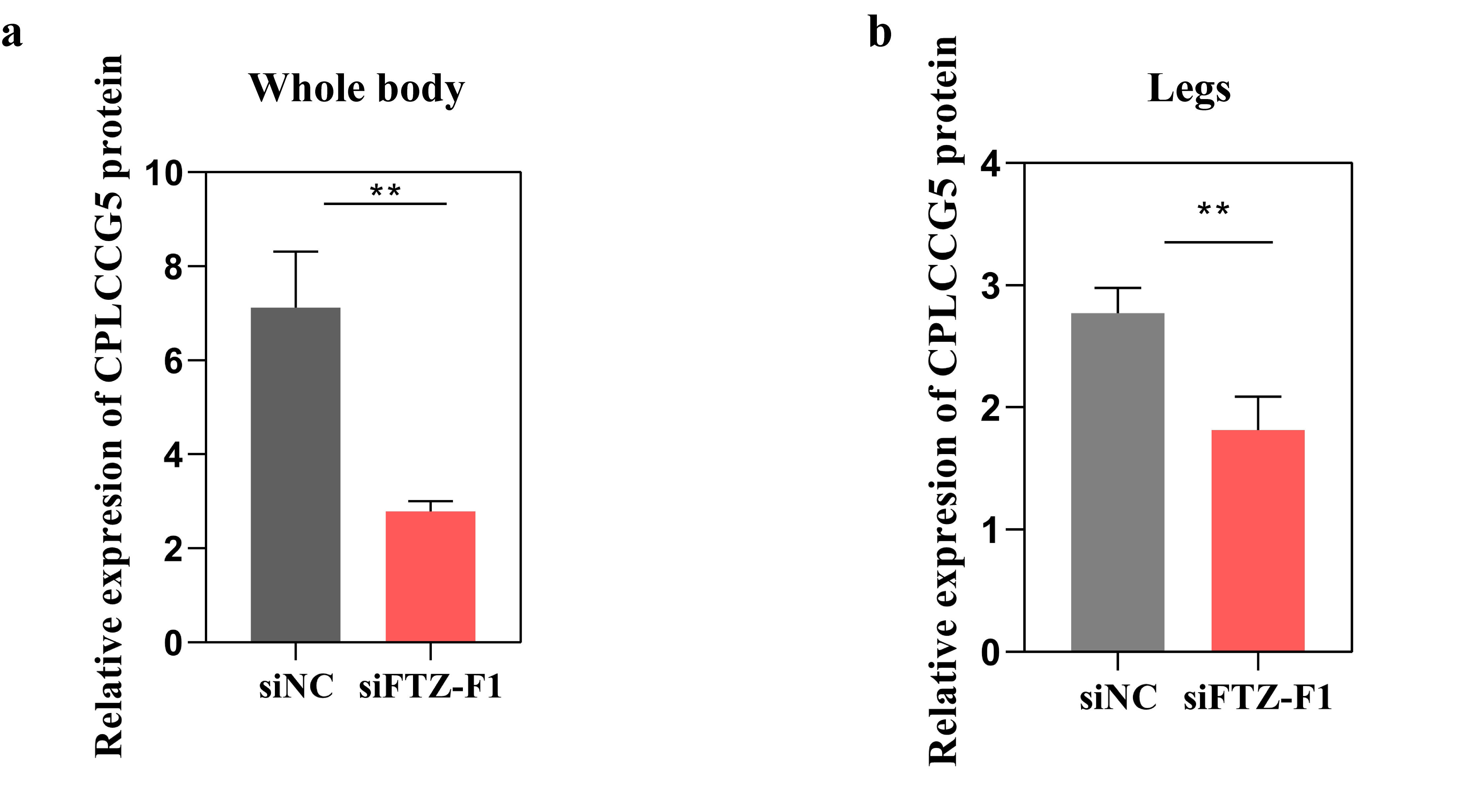

Supplement: Supplementary file 3 — Additional file 3: Figure S1. a The expression analysis of CPLCG5 protein in mosquito’s whole body after silencing of FTZ-F1, as assessed using IPP software. b The expression analysis of CPLCG5 protein in mosquito’s leg after silencing of FTZ-F1, as assessed using IPP software. The results are shown as the mean ± SD of 3 biological replicates. (P < 0.01**). [file 13071_2020_4383_MOESM3_ESM.tif]

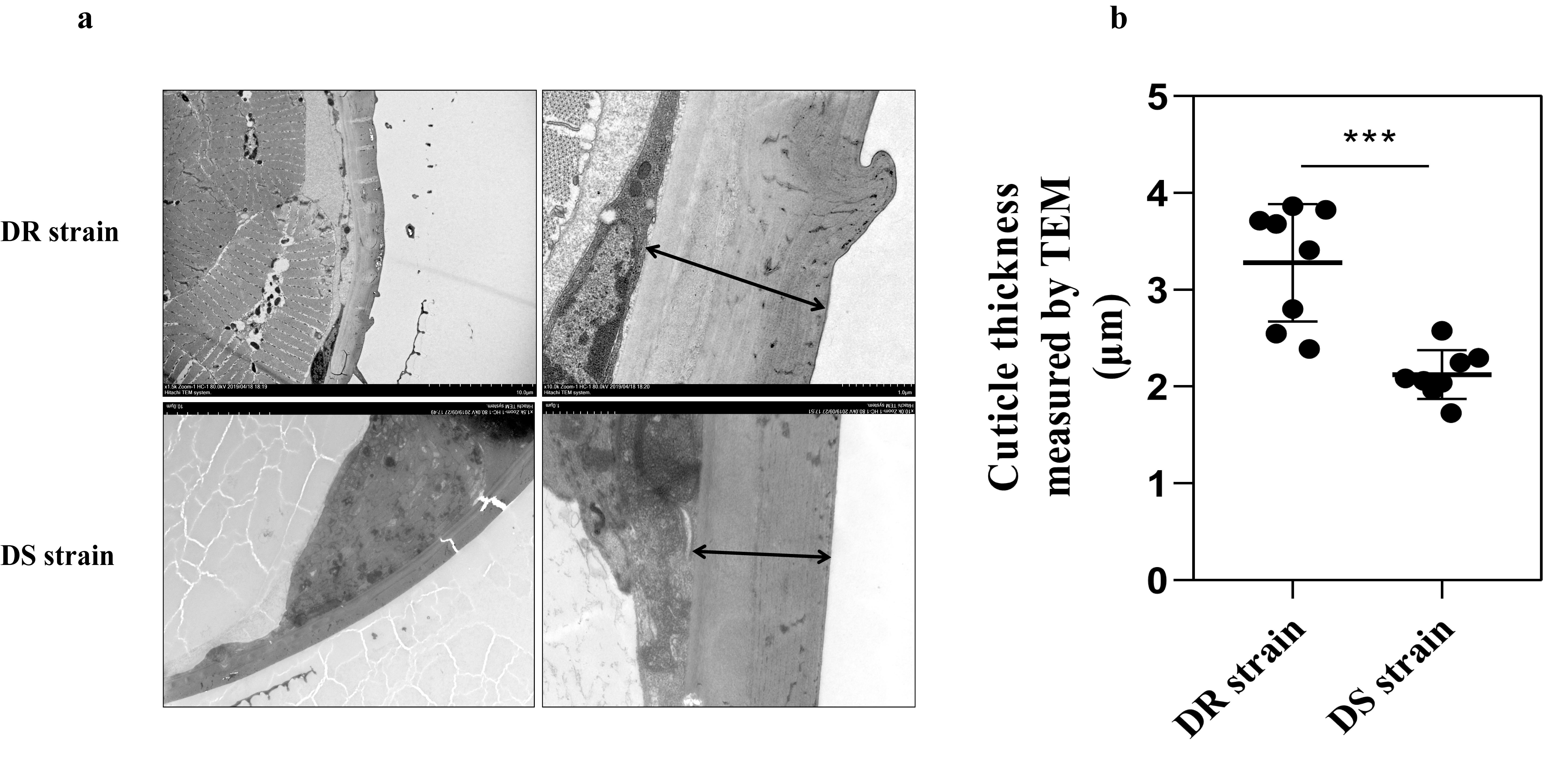

Supplement: Supplementary file 4 — Additional file 4: Figure S2. a TEM analysis of the ultrastructure of mosquito tarsi cuticles in the DR strain and DS strain. The black double arrow indicates the cuticle thickness. b Scatter plot of the cuticle thickness of the DR strain and DS strain. ***P ≤ 0.001. Abbreviation: n = the number of measures taken for each batch of 8 mosquitoes (siNC & siFTZ-F1). [file 13071_2020_4383_MOESM4_ESM.tif]
